# Vagus nerve stimulation for chronic pain management: Mechanisms and clinical advances

**DOI:** 10.14440/jbm.0051

**Published:** 2025-10-08

**Authors:** Milan Patel, Shahnaz Fooladi Sarabi, Kalvin Chen, Lukas J. Henjum, Barnabas T. Shiferaw, Danielle Kohr, Alaa Abd-Elsayed

**Affiliations:** 1 Department of Anesthesiology, School of Medicine and Public Health, University of Wisconsin-Madison, Madison, Wisconsin 53706, United States of America; 2 Department of Anesthesiology, Critical Care Medicine, School of Medicine, Ardabil University of Medical Sciences, Ardabil 55136, Iran; 3 Department of Pain Medicine, Tertiary Referral Clinic Löwenstein, Löwenstein, Baden-Württemberg 74245, Germany

**Keywords:** Chronic pain, Vagus nerve stimulation, Headache, Migraine, Inflammation, Neuropathy

## Abstract

**Background:**

Chronic pain poses a significant challenge to the general population, especially in older adults. As a result, there is a growing emphasis on developing novel treatment options and expanding the use of emerging technologies.

**Objective:**

This comprehensive review aims to guide practitioners in chronic pain management by extending the current application of vagus nerve stimulation (VNS). The review examined peer-reviewed studies, including multicenter cohort studies and clinical trials, published within the past 25 years. VNS has shown the most promising results in pain reduction for headache and migraine, with less relief in inflammatory and neuropathic pain as compared to current first-line treatment options.

**Conclusion:**

Overall, this review seeks to compile and critically analyze the effect and efficacy of VNS on different forms of chronic pain using the most current studies and research applicable.

## 1. Introduction

The vagus nerve, cranial nerve 10 (CN X), is the longest of the CN. It spans the distance between the medulla and the colon, innervating the thoracic and abdominal portions of the body. The vagus nerve plays a key role in multiple systems, including the autonomic, cardiovascular, respiratory, gastrointestinal, immune, and endocrine systems.^1^ A key function of the vagus nerve is its interaction in brain-body communication. Brain–body communication is integral for maintaining proper function and overall health of the individual. The vagus nerve facilitates brain-body communication by modulating inflammatory and immune responses.^2^

The vagus nerve comprises sensory, motor, and parasympathetic fibers, giving it particular significance in chronic pain management. Approximately 80% of its fibers are afferent and 20% are efferent. They are classified into three types—A, B, and C—according to Erlanger and Glasser, based on their conduction velocities related to their respective sizes.^4^ A-type fibers are myelinated; the larger subtype transmits somatic afferent and efferent information, while the smaller A subtype primarily carries visceral afferent information. B-type fibers contribute to efferent sympathetic activity, providing innervation to the parasympathetic preganglionic areas. C-type fibers are the smallest and unmyelinated, predominantly transmitting visceral afferent information; notably, they account for 60–80% of all vagus nerve fibers.

Current pain management stimulation options aim to stimulate the peripheral nervous system and central nervous system (CNS), with room for improvement. Pharmacological intervention over a long period can produce undesirable side effects, while interventional procedures may lose efficacy prematurely in some cases. Thus, exploration into vagus nerve stimulation (VNS) is necessary.

This review examines the potential of VNS to alter the course of chronic pain treatment.^3^ It begins with an understanding of the anatomy and physiology of the vagus nerve, allowing for broader applications and novel therapies. Subsequently, the mechanisms of VNS are explored, emphasizing its role in inflammatory pathways and neuroinflammation. Furthermore, VNS is evaluated across its common clinical applications, including neuropathic pain, inflammatory pain, headache, and migraine. Expanding on the current literature within these fields provides an excellent foundation for future studies and enhances overall understanding.

## 2. Mechanisms of VNS in pain regulation

VNS is thought to ameliorate and regulate pain through several mechanisms.^5^ A key pathway is the cholinergic anti-inflammatory pathway (CAP).^6^-^9^ Activation of the vagus nerve triggers the release of acetylcholine, which binds to alpha-7 nicotinic acetylcholine receptors (α7nAChR) on macrophages, leading to downstream inhibition of pro-inflammatory cytokines, including tumor necrosis factor-alpha (TNF-α), interleukin (IL)-1 beta, and IL-6.^10^,^11^ Through the mediation of these systemic inflammatory mediators, VNS can reduce pain associated with chronic inflammatory conditions, such as rheumatoid arthritis and fibromyalgia.

In addition to cytokine modulation, VNS alters pain perception by modulating neurotransmitters. It increases the release of norepinephrine and serotonin in the CNS, both of which are implicated in the modulation of pain perception.^12^-^14^ VNS also reduces glutamate excitotoxicity, lowering oxidative stress, cell death, hyperalgesia, and allodynia.^15^-^17^ Furthermore, it has been demonstrated to suppress microglial activation and reduce neuroinflammation through α7nAchR, further contributing to its analgesic effects.^18^ Moreover, VNS alters pain perception by engaging key pain-regulatory structures in the CNS, including the periaqueductal gray, locus coeruleus, and nucleus tractus solitaries.^18^-^20^ Through these mechanisms, VNS presents a promising neuromodulatory approach for managing chronic pain.

Notably, transcutaneous auricular VNS (taVNS), a non-invasive approach, has demonstrated therapeutic potential in drug-resistant migraine, treatment-resistant depression, and tinnitus, as reflected in its approval by the United States food and drug administration (FDA).^20^

## 3. Clinical applications of VNS for chronic pain

This chapter outlines the clinical applications of VNS in the treatment of chronic pain. VNS has proven effective in treating various types of pain, including neuropathic pain, inflammatory pain, headaches, and migraine. Despite these promising results, further research is needed to enhance treatment outcomes.

### 3.1. Neuropathic pain

An open-label Phase I/II trial study conducted by Lange *et al*.^12^ investigated the efficacy and overall safety of VNS in patients with treatment-resistant fibromyalgia. The study consisted of 14 patients initially implanted with the VNS device. Twelve patients completed the initial 3-month evaluation of VNS efficacy, and 11 patients were followed up at 5, 8, and 11 months. Overall, therapeutic efficacy was the primary measure, with pain relief, wellness, and functionality also evaluated. The side effects observed in this experimental group were consistent with previous studies conducted with fibromyalgia patients. While VNS could be a potential solution with significant efficacy, initial results suggest that its side effects and tolerability are comparable to current mainstream options. Further research could be conducted on a larger scale to test VNS efficacy in a larger sample.

According to a study conducted by Kutlu *et al.*,^21^ the relationship between auricular VNS (aVNS) and an exercise program on the quality of life (QoL) of fibromyalgia patients was investigated. The study included 60 female participants, randomly assigned to two groups of 30. One group performed a set of 20 home-based exercises, while the other performed the same exercises paired with aVNS. Baseline assessments were conducted, and outcomes were re-evaluated at study completion using a visual analog scale (VAS), the Beck Depression Inventory, the Beck Anxiety Inventory, the Fibromyalgia Impact Questionnaire, and the Short Form-36. The study reported that both groups demonstrated significant improvements in pain, depression, anxiety, functionality, and QoL scores. While VNS did not appear to provide a significant additive effect overall, the Short Form-36 was the sole contributor to show a significant difference between groups, suggesting a potential QoL benefit with VNS. Although the VNS group exhibited greater improvements compared to the exercise-only group, these differences were not statistically significant.

Muthulingam *et al.*,^22^ evaluated the anti-nociceptive potential of transcutaneous VNS (tVNS) in patients with chronic pancreatitis using a randomized double-blind crossover trial with both sham and active stimulation. Participants were randomly given 2-week timeframes of cervical tVNS followed by sham stimulation, or vice versa. Outcomes included overall pain relief, the global impression of change score, QoL, and the Brief Pain Inventory questionnaire. Similar to previous studies, results showed minimal to no differences between tVNS and sham, with neither primary nor secondary endpoints achieved. These findings suggest that further research is needed in this area. Given its stronger evidence base in headache and migraine, the limited efficacy observed here may reflect the challenges of applying VNS to neuropathic pain.

### 3.2. Inflammatory pain

Initial studies have shown the important role the vagus nerve plays in inflammatory responses and chronic pain. Specifically, the CAP plays a critical role in mediating the anti-inflammatory properties of the vagus nerve.^4^

Farmer *et al.*,^23^ investigated the effects of tVNS on acid-induced esophageal pain. The study involved a 30-min infusion of 0.15 M hydrochloric acid into the distal portion of the esophagus. In one arm, 15 healthy participants were randomly assigned to receive either tVNS or sham treatment during the acid infusion. A second group of 18 healthy participants underwent a randomized crossover design with both tVNS and sham. Both groups demonstrated pain reduction with tVNS compared to sham, suggesting potential efficacy of tVNS in treating esophageal pain.

Shi *et al.*,^24^ further examined VNS in patients with irritable bowel syndrome (IBS). Forty-two participants were randomly assigned to either sham treatment or tVNS for 4 weeks. Outcomes included abdominal pain (measured through VAS), anorectal motor and sensory function (assessed with high-resolution anorectal manometry), and autonomic function (through electrocardiogram). Results showed significant improvements with tVNS, including reduced VAS pain scores, improved QoL, decreased IBS symptoms, enhanced anorectal inhibitory reflex, and improved rectal sensation.

Taken together, these studies support the potential of tVNS in inflammatory pain conditions. In IBS, tVNS alleviated both constipation and abdominal pain, further highlighting its therapeutic promise.

### 3.3. Headache and migraine

In an open-label study conducted by Goadsby *et al.*,^25^ the efficacy of non-invasive VNS (nVNS) in migraine treatment was evaluated. Participants had up to four migraines treated with two 90-s doses administered 15 min apart, over 6 weeks. VNS was delivered to the right cervical branch of the vagus nerve. Pain intensity was rated after treatment, ranging from moderate to severe. Among the 30 participants, 13 reported adverse events, including neck twitching, raspy voice, or redness at the site of the device. Notably, no unanticipated or adverse events were reported. Of the 19 events in participants who treated their first migraine at baseline, four reported no pain at the 2-h mark. In cases rated moderate to severe at baseline, the pain-free rate was slightly higher at 12 out of 54 events.

Barbanti *et al.*,^26^ conducted a multicenter open-label study to further evaluate nVNS as a treatment option for migraine. Patients with high-frequency episodic migraine (HFEM) and chronic migraine were treated with VNS for up to three consecutive migraine attacks over 2 weeks. nVNS was administered to the right cervical branch of the vagus nerve in two 120-s doses, delivered 3 min apart. Of the 50 enrolled patients, 48 were treated for a final count of 131 migraine events. Based on VAS scores, pain relief >50% was achieved in 56.3% of patients at 1 h and 64.6% at 2 h. Pain-free status was reported by 35.4% of patients at 1 h and 39.6% at 2 h. These findings suggest that nVNS is an effective therapeutic option in treating migraines.

Silberstein *et al.*,^27^ conducted a multicenter, double-blind study to evaluate the safety and efficacy of nVNS in adults with chronic migraine. After a 1-month baseline measurement, participants were randomly assigned to receive either nVNS or sham treatment for 2 months. The primary outcomes were safety, tolerability, and changes in headache days within 28 days, accounting for acute medication usage. A total of 59 participants were enrolled. Tolerability was comparable between groups, with most adverse events not causing any significant pain. The mean reduction in headache days was 1.4 with VNS compared to 0.2 with sham. Based on the 15 open-label phase completers in the VNS category, the mean reduction in headache days from baseline was 7.9 after 8 months of VNS. These findings suggest that VNS is effective in treating chronic migraine and headache. However, additional studies are necessary to validate these results.

Najib *et al.*,^28^ further investigated the potential of nVNS in migraine prevention through a double-blind, sham-controlled study. Participants were monitored for 12 weeks, with 336 participants enrolled. Of these, 113 completed at least 70 days of the protocol and adhered to instructions for at least 66% of the total duration. COVID-19 impacted the original study, significantly reducing the intended study duration and sample size. Results showed an average reduction of 3.12 monthly migraine days in the VNS group compared to 2.29 days in the sham group. Furthermore, the responder rate was also higher with VNS at 44.87%, versus 26.81% in the sham group. No device-related adverse events were reported. Overall, these findings support the growing evidence for nVNS as a promising approach in treating migraine-related chronic pain. However, more robust studies are needed to confirm efficacy. A breakdown of the key pain types and outcomes from the studies discussed is summarized in Table 1 to facilitate comparison across studies.

**Table 1:** Overview of clinical studies on vagus nerve stimulation for chronic pain

Study	Pain type	Key outcomes
Lange *et al*.^12^	Neuropathic	Similar results to existing treatment options; positive results indicated
Kutlu *et al*.^21^	Neuropathic	Both home-based exercise and the same exercise paired with auricular VNS groups showed an improvement trend, with only the Short Form-36 results demonstrating significant benefits
Muthulingam *et al*.^22^	Neuropathic	No significant difference between tVNS and sham treatments
Farmer *et al*.^23^	Inflammatory	Pain reduction observed in tVNS groups compared to sham
Shi *et al*.^24^	Inflammatory	tVNS improved VAS, QoL, and rectal sensation; effective for irritable bowel syndrome
Goadsby *et al*.^25^	Migraine	No serious or unexpected events occurred; 4/19 mild cases reported no pain at the 2-h mark; 12/54 moderate and severe cases reported pain-free
Barbanti *et al*.^26^	Migraine	64.6% VAS reduction at 2 h; 39.6% pain-free rate
Silberstein *et al*.^27^	Migraine	Mean reduction of 1.4 headache days in the VNS group versus 0.2 days in the sham group; good tolerability
Najib *et al*.^28^	Migraine	Mean reduction of 3.12 headache days in the VNS group versus 2.29 days in the sham group; higher responder rate

Abbreviations: QoL: Quality of life; tVNS: Transcutaneous vagus nerve stimulation; VAS: Visual analog scale; VNS: Vagus nerve stimulation.

## 4. Current evidence and clinical trials

This section reviews VNS trials, highlighting its positive effects in treating headaches, including episodic, cluster, and migraine types, as well as its demonstrated effectiveness in treating stroke-related complications.

nVNS has been used in the treatment of episodic cluster headache and chronic cluster headache. In one study, VNS-treated patients with episodic cluster headache experienced a significant reduction in pain compared to baseline.^29^ In contrast, patients with chronic cluster headache showed no notable improvement after 15 min. However, limitations of this study included the short 15-min measurement window, which may have underestimated treatment effects, and the lack of efficacy in chronic cluster headache.

In patients with HFEM and chronic migraine, nVNS also demonstrated substantial results. After 2 h of stimulation, 50% of patients with HFEM and 26.5% of patients with chronic migraine were pain-free.^30^ However, limitations of this study included the small sample size (*n* = 50) and differences in headache severity, with chronic migraine typically reported as mild-to-moderate and episodic headaches as severe.

Another study assessed tVNS in patients with post-COVID-related headaches and found significant reductions in VAS scores, suggesting potential benefit.^30^ Similarly, limitations of this study included a small sample size (*n* = 30). In addition, tVNS has been shown to reduce pain sensitivity, lowering tonic heat pain and increasing mechanical pain thresholds.^31^ More studies on tVNS are necessary to confirm its efficacy.

Studies by Lindemann *et al.*,^32^ and Hays *et al.*,^33^ investigated the efficacy of VNS on cortical spreading depolarization related to stroke and loss of forelimb function. VNS has been observed to be effective in treating both. In a rat model of focal ischemia, invasive VNS reduced the frequency of spreading depolarization without affecting pulse rate, respiratory rate, or oxygen saturation. In addition, pairing VNS with rehabilitation markedly improved forelimb recovery. While rehabilitation alone restored forelimb recovery to 34 ± 19%, combining it with VNS increased recovery to 96 ± 3%. These findings suggest that VNS may be an effective treatment for elderly stroke patients, both in reducing the frequency of spreading depolarization and in enhancing forelimb function.

Moreover, VNS has demonstrated clinical benefits in treating drug-resistant depression and refractory epilepsy. Mechanistically, VNS increases hippocampal norepinephrine concentrations, which may contribute to its therapeutic effects. This application has shown success in treating depression and epilepsy.^34^ However, one study revealed significant limitations in its double-blind design. In the sham group and the nVNS group, there were reductions in the number of headache days per month, suggesting that the sham device functioned as a strong placebo. In fact, sham stimulation often produced greater symptom relief than conventional placebo pill. Treatment allocation was correctly guessed by 58% of patients in the VNS group and 62% in the sham group.^35^ A recurring limitation of these studies is the small sample size (*n* = 30–50), which makes it difficult to establish statistical significance. Future research with larger cohorts and improved placebo controls is essential to confirm the efficacy of VNS and reduce confounding placebo effects.

## 5. Future directions and innovations

Future innovations and areas requiring improvement are discussed in this chapter. While VNS has proven effective in treating various chronic conditions, there remains significant potential for advancement. Emerging directions include the development of personalized stimulation protocols and the combination of VNS with other therapeutic modalities.

### 5.1. Emerging technologies and wearable VNS devices

Innovations in precision neuromodulation and advancements in device design have the potential to improve therapeutic efficacy and patient compliance. The non-invasive nature of VNS broadens its applicability and facilitates its use across diseases. Traditional nVNS, such as tVNS, uses skin electrodes for stimulation and offers a non-invasive alternative to surgically implanted VNS devices, which are limited by their invasive nature.^36^,^37^ The primary application areas for nVNS are the auricular and cervical regions.^38^ Compared to implanted systems, nVNS carries a lower risk of adverse effects, including infection, post-surgical complications, implant rejection, and patient pain. Furthermore, taVNS is inexpensive, safe, and portable, enhancing its feasibility for clinical translation and widespread adoption.^39^,^40^

Recent advances in nVNS technology have introduced real-time feedback mechanisms that assess vital physiological markers and adjust stimulation parameters to optimize therapeutic efficacy.^41^ Clinically, nVNS is utilized for various neurological conditions, including chronic migraines, headaches, depression, epilepsy, and pain management.^42^-^45^ It has also shown beneficial effects in chronic pain diseases, such as systemic lupus erythematosus-related pain and IBS.^46^,^24^ Miniaturization and enhanced portability of these devices enable continuous or on-demand therapy tailored to individual needs, making nVNS a more practical treatment option.^48^ Its wearable design further increases accessibility for clinicians and patients, paving the way for exploring VNS in new contexts, such as at-home treatment.^49^,^50^ While nVNS, especially taVNS, has significant potential for broader adoption, advancements in device engineering and investigation of its underlying mechanisms are essential to achieve its aims in neurorehabilitation.^40^

### 5.2. Potential for personalized VNS therapy

Developing personalized VNS paradigms requires an understanding of the targeted disorders.^45^,^47^,^50^,^51^ Advances in precision neuromodulation are driving nVNS toward more effective treatments.^52^ Neuromodulation, particularly electrical stimulation of the autonomic nervous system, shows promise as a non-pharmacological approach for various chronic conditions.^53^-^55^ Precision VNS selectively stimulates different vagus nerve fiber bundles, allowing precise control over heart rate regulation while minimizing off-target effects.^56^-^58^

Non-invasive methods, such as aVNS, are gaining popularity due to their safety profile and lower costs.^59^-^62^ aVNS has proven effective for conditions such as migraines and chronic pain, with fewer adverse effects than traditional methods.^56^,^63^ However, the therapeutic outcomes of aVNS can be inconsistent and unpredictable. The necessary dose for treatment is not specified, and issues of over- and under-stimulation can lead to failure to respond. The parameters are often chosen empirically based on patient feedback.^64^-^67^

To address this challenge, a closed-loop aVNS system, which continuously personalizes stimulation based on real-time biofeedback, is necessary to meet individual physiological demands.^68^ Such systems adjust settings based on autonomic biomarkers, such as heart rate variability and respiration.^69^,^70^ Personalized VNS can enhance the durability of positive treatment effects, minimize adverse effects, and lower the energy footprint of stimulation patterns, ultimately reducing the number of treatment-resistant patients.^71^ Advances in technology are enhancing these systems by integrating real-time adjustments based on patient data.^72^ As precision neuromodulation evolves, it is poised to make aVNS a key component in personalized medicine, with expanding applications across inflammatory and neurological disorders.^48^ Despite its safety advantages, questions remain regarding the efficacy and neurophysiological mechanisms underlying VNS.^44^,^58^

### 5.3. Combination therapies

Combining nVNS with complementary treatment strategies can enhance treatment effects in complex inflammatory diseases, improve autonomic balance, and manage chronic inflammation. When paired with methods such as transcranial magnetic stimulation or transcutaneous electrical nerve stimulation,^73^,^75^ nVNS demonstrates greater synergistic potential and longer-lasting effects compared to either treatment alone. In addition, this combined approach has been especially promising for headaches and migraines.^4^

In rehabilitation contexts, pairing VNS with sensory and motor stimulation significantly enhances neuroplasticity and strengthens personalized treatment efficacy.^75^ By rapidly engaging neuromodulatory networks, VNS boosts training-related synaptic plasticity^76^ and facilitates recovery in conditions such as spinal cord injury, brain injury, and peripheral nerve damage.^77^-^81^ These synergistic effects have contributed to the FDA’s approval of VNS for stroke rehabilitation.^82^

Beyond physical recovery, VNS combined with cognitive behavioral therapy has shown benefit in improving psychological conditions, including depression.^81^ The underlying mechanism involves modulation of neurochemical pathways that support neuroplasticity and enhance cognitive functions.^82^ Future investigation is necessary to determine the optimal treatment durations, indications, and individualized protocols to maximize the benefits of VNS in multimodal therapeutic strategies.^41^

Rehabilitation exercises paired with VNS for stroke patients have traditionally been standardized for clinical trials, but innovative strategies such as telerehabilitation and game-based exercises also show promise.^84^ Moreover, combining dietary interventions with VNS has demonstrated synergistic effects, suggesting that longer off-times in VNS may enhance effectiveness.^85^ Combining VNS with pharmacological agents, such as anti-inflammatory medications or biologics, has also shown positive results, such as amplifying the effects of TNF inhibitors in rheumatoid arthritis and reducing corticosteroid doses in asthma, thereby improving patient outcomes.^86^ A summary of these combinatorial methods and their key findings is presented in Table 2, highlighting which approaches yielded the most promising results.

**Table 2:** Overview of combinatorial vagus nerve stimulation (VNS) therapies and key findings

Study	Combination strategy	Key findings
Holland *et al*.^71^ Sackeim^72^	Non-invasive VNS+TMS or TENS	Potential to enhance neuroplasticity, autonomic balance, and chronic inflammation management
Hays *et al*.^73^ Hays *et al*.^74^ Khodaparast *et al*.^75^ Hays *et al*.^76^ Meyers *et al*.^77^ Noble *et al*.^78^ Hays *et al*.^79^ De Ridder *et al*.^80^ Tyler *et al*.^81^	VNS+specific training (rehabilitation)	• Stimulates neuromodulatory networks and boosts synaptic plasticity related to training • Helps with recovery in spinal cord injury, brain injury, and peripheral nervous system damage • Led to the FDA’s approval of VNS for stroke treatment
Abd-Elsayed *et al*.^82^ Engineer *et al*.^83^	VNS+CBT	Improvement in psychological conditions, such as depression, through neurochemical activity
Kossoff *et al*.^85^	VNS+dietary interventions	• Displays synergistic effects • Longer off-times may increase VNS effectiveness
Sauer *et al*.^86^	VNS+pharmacological agents or biologics	Amplifies anti-inflammatory drug effects, reduces corticosteroid dose, and improves outcomes

Abbreviations: CBT: Cognitive behavioral therapy; FDA: Food and drug administration; TENS: Transcutaneous electrical nerve stimulation; TM%nscranial magnetic stimulation.

## 6. Conclusion

This review highlights the potential of VNS as an effective strategy for alleviating chronic pain. VNS has demonstrated therapeutic benefits in conditions such as rheumatoid arthritis, fibromyalgia, diabetic neuropathy, and migraines, representing a revolutionary technique in chronic pain management. Its analgesic effects are thought to be mediated through diverse pathways, including the CAP, neurotransmitter modulation, and suppression of microglial activation. nVNS has been effective in alleviating pain in episodic migraines, cluster headaches, and COVID-19-related headaches, though its efficacy in chronic cluster headaches requires more research. Experimental evidence also supports its role in reducing pain sensitivity and enhancing recovery in stroke models. The non-invasive characteristic of aVNS may broaden its applicability across a wider range of diseases, while innovations in engineering, precision neuromodulation, and equipment development continue to improve therapeutic efficacy.

Despite these advances, challenges remain. Achieving consistent outcomes and determining optimal dosing remain obstacles, particularly in aVNS. Closed-loop aVNS systems that personalize stimulation in real time using autonomic markers, such as heart rate and respiratory rate, are essential to meet individual physiological needs. nVNS represents a promising step toward wearable devices for personalized therapy. However, challenges such as limited sample size, placebo effects, and contradictory results highlight the need for further research. Overall, VNS offers encouraging outcomes for chronic pain management. Continue research into combination therapies and technological innovations is essential to maximize the potential of VNS and establish novel, effective approaches for managing chronic and complex pain conditions.

## Data Availability

Not applicable.
